# Complicaciones en pacientes usuarios de traqueostomía en unidades de cuidados intensivos. Scoping Review

**DOI:** 10.15649/cuidarte.2281

**Published:** 2023-03-31

**Authors:** Mabel Magoth Reyes-Pulido, Mauricio Orozco-Levi, Alba Lucía Ramírez-Sarmiento, Angelica Julieth Nariño-Gamboa, Andry Giseth Fragozo-Ibarra

**Affiliations:** 1 . Universidad de Santander UDES, Bucaramanga, Colombia. Email: ma.reyes@mail.udes.edu.co Universidad de Santander Universidad de Santander Colombia ma.reyes@mail.udes.edu.co; 2 . Fundación Cardiovascular- Hospital Internacional de Colombia- Servicio de Neumología Centro para el Cuidado de la Salud Respiratoria, Bucaramanga, Colombia Email: mauricioorozco@fcv.org Fundación Cardiovascular- Hospital Internacional de Colombia- Servicio de Neumología Centro para el Cuidado de la Salud Respiratoria Colombia mauricioorozco@fcv.org; 3 . Fundación Cardiovascular- Hospital Internacional de Colombia- Servicio de Neumología Centro para el Cuidado de la Salud Respiratoria, Bucaramanga, Colombia. Email: alba.ramirez.sarmiento@gmail.com Fundación Cardiovascular- Hospital Internacional de Colombia- Servicio de Neumología Centro para el Cuidado de la Salud Respiratoria Colombia alba.ramirez.sarmiento@gmail.com; 4 . Universidad de Santander UDES, Bucaramanga, Colombia. Email: uniangelica21@hotmail.com Universidad de Santander Universidad de Santander Colombia uniangelica21@hotmail.com; 5 . Universidad de Santander UDES, Bucaramanga, Colombia. Email: andris fragozo@hotmail.com Universidad de Santander Universidad de Santander Colombia fragozo@hotmail.com

**Keywords:** Traqueostomía, Complicaciones Intraoperatorias, Complicaciones Postoperatorias, Cuidados Críticos., Tracheostomy, Intraoperative Complications, Postoperative Complications, Critical Care., Traqueostomia, Complicates Intraoperatórias, Complicates Pós-Operatórias, Cuidados Críticos.

## Abstract

**Introducción:**

Los procedimientos de traqueostomía van en aumento en las unidades de cuidados intensivos en el adulto, por lo que las complicaciones asociadas a este procedimiento también incrementan. Se ha identificado que la traqueostomía puede conllevar a complicaciones tanto preoperatorias como postoperatorias, que varían ampliamente entre un 5-40%, entre las más frecuentes están, estenosis traqueal, fístula traqueoesofágica, hemorragia, lesión peristomal, decanulación, infecciones, entre otras, que podrían causar la muerte hasta en 1,4% de las personas. Sin embargo, a nivel mundial muy pocos estudios abordan los conceptos causales o factores de riesgo mecánicos y no mecánicos de este importante tema.

**Objetivo:**

Revisar el alcance de la literatura científica disponible sobre las complicaciones de origen mecánico y no mecánico asociadas a la traqueostomía en pacientes adultos en las unidades de cuidados intensivos entre el periodo 2015-2020.

**Materiales y métodos:**

se establecieron la pregunta de investigación con metodología “Patient, Intervention, Comparation, Outcome, Time (PICOT)” y los criterios de inclusión para la búsqueda de los referentes bibliográficos de estudios observacionales y experimentales. La información fue consultada en las bases de datos PubMed y EBSCO y los artículos científicos seleccionados fueron los publicados entre los años 2015-2020. Como guía metodológica y de calidad para el presente estudio se utilizó la lista de chequeo PRISMA-ScR.

**Resultados:**

las complicaciones con mayor frecuencia son: sangrado 61% presentada (13/21 artículos), estenosis traqueal 28,5% (5/21 artículos), decanulación 23,6% (5/21 artículos), infección de la estoma 19% (4/21 artículos) muerte 19% (4/21) y la dificultad en la inserción de la cánula 19% (4/21 artículos), en cuanto a factores de riesgo mecánicos para éstas sólo se identifica el uso de la técnica Bjork flap (OR=0,4). Entre los no mecánicos se encontraron, obesidad (OR=5,15), diámetro de cánula >6 (OR= 2,6) y ventilación mecánica preoperatoria (OR=3,14).

**Conclusiones:**

Se logró identificar que las complicaciones relacionadas con la traqueostomía con mayor incidencia son sangrado, estenosis traqueal, decanulación accidental y la muerte. Sin embargo, aún se desconoce si se originan por una causa mecánica o no mecánica durante su manejo en UCI.

## Abstract (en)

**Introduction:**

Tracheostomy procedures in intensive care units are on the rise; however, they can lead to both perioperative and postoperative complications, with a variable incidence from 5 to 40% and even death in up to 1.4% of individuals. Despite this, few studies address causal concepts or mechanical and non mechanical risk factors about this important topic.

**Objective:**

To review the scope of the available scientific literature on complications of mechanical and non-mechanical origin associated with a tracheostomy.

**Materials and Methods:**

The research question and inclusion criteria were established to conduct the search in PubMed and EBSCO databases between 2015 and 2020. The PRISMA-ScR checklist was used in the present study as a methodological and quality guideline.

**Results:**

The most frequent complications were bleeding 61%, tracheal stenosis 28.5%, decannulation 23.6% (5/21) , stoma infection 19%, death 19%, and difficult tracheostomy tube insertion 19%. Regarding mechanical risk factors, only the use of the Bjork flap (OR=0.4) was identified as a protective factor. Among the non-mechanical factors, obesity (OR=5.15), tube diameter >6 (OR=2.6), and preoperative mechanical ventilation (OR=3.14) were found.

**Conclusions:**

It was possible to identify that the highest incidence of tracheostomy-related complications were bleeding, tracheal stenosis, accidental decannulation, and death; however, it is still unknown whether they originate from a mechanical or non-mechanical cause during intensive care management.

## Resumo

**Introdujo:**

Os procedimentos de traqueostomia estao aumentando nas unidades de terapia intensiva, entretanto, podem levar a complicates perioperatórias e pós-operatórias com uma incidencia variável entre 5-40% e até a morte em até 1,4% dos indivíduos, apesar disso, poucos estudos abordam os conceitos causais ou fatores de risco mecánicos e nao-mecánicos sobre este importante tópico.

**Objetivo:**

Rever o escopo da literatura científica disponível sobre complicates mecánicas e nao mecánicas associadas a traqueostomia.

**Materiais e métodos:**

a questao da pesquisa e critérios de inclusao foram estabelecidos para a pesquisa nas bases de dados PubMed e EBSCO entre os anos 2015-2020, como guia metodológico e de qualidade para o presente estudo foi utilizada a lista de verificagao PRISMA- ScR.

**Resultados:**

As complicates com maior freqüencia foram, sangramento 61%, estenose traqueal 28,5%, decanulagao 23,6% (5/21, infecto por estoma 19%, morte 19% e dificuldade na inserto da cánula 19%, em relato aos fatores de risco mecánico, apenas o uso da técnica de Bjork Flap (OR=0,4) foi identificado como fator de proteto, entre os fatores nao mecánicos foram encontrados: obesidade (OR=5,15), diámetro da cánula >6 (OR= 2,6) e ventilagao mecánica pré-operatória (OR=3,14).

**Conclusóes:**

Conseguimos identificar que as complicates relacionadas a traqueostomia com maior incidencia foram sangramento, estenose traqueal, decanulagao acidental e morte, no entanto, ainda nao se sabe se elas se originam de uma causa mecánica ou nao mecánica durante a gestao dos cuidados intensivos.

## Introducción

La Traqueostomía (TQT) es de las técnicas más frecuentemente usadas en Unidades de Cuidado Intensivo (UCI) para disminuir la instancia hospitalaria de las personas con soporte ventilatorio, asegurar la vía aérea como procedimiento de emergencia ante una obstrucción causada por algún trauma y reducir las infecciones asociadas a la Ventilación Mecánica (VM1). Este procedimiento se asocia a una mejoría significativa del paciente, rehabilitación temprana, reducción de los requerimientos de sedación, analgesia y tiempo de estancia en la UCI^2^^-^^4^. A nivel mundial, se prevé un aumento del 80% de la necesidad de VM para el año 2026^5^, lo que sugiere en consecuencia un crecimiento en la frecuencia del uso de la TQT en las UCI alrededor del mundo, lo cual a su vez requiere apoyo profesional interdisciplinario con formación y experticia en su manejo^6^.

La Organización Mundial de la Salud (OMS) estima que los índices de complicaciones en pacientes con TQT y en instancia hospitalaria son elevados, con una probabilidad entre el 5% y 10% de desarrollar una infección nosocomial por TQT^7^, siendo de las complicaciones más prevalentes en pacientes con estancia hospitalaria prolongada. Un estudio realizado en hospitales de España reportó que durante la realización del procedimiento de TQT las complicaciones encontradas fueron, 20% hemorragia, 19% dificultad en la inserción de la cánula, 4% atelectasias, 1% desaturación y 1% ruptura de anillo traqueal; posterior al procedimiento un 15,3% presentó estenosis traqueal y un 5% paresia de cuerdas bocales, en total un 65,3% de los pacientes que participaron en el estudio presentaron algún tipo de complicación asociada al procedimiento de TQT^8^.

A nivel de América Latina en un estudio realizado en el 2014 en Brasil se reportó que la prevalencia de la morbilidad asociada a TQT varía entre de un 4% a un 10%, siendo la principal complicación la hemorragia en el período postoperatorio con un 3,7%, seguido por la obstrucción de la cánula por secreciones con el 2,7% y el desplazamiento de la cánula un 1,5%^9^.

En Colombia existen pocos estudios publicados sobre la prevalencia e incidencia de las complicaciones debido al uso de la TQT, uno de ellos es el publicado por Calvache y colaboradores en el año 2013 realizado en la clínica La Estancia en Popayán que reportó que un 1,6% de las complicaciones se presentaron en fase intraoperatoria y un 9,1% en postoperatoria, y que de acuerdo al valor predictivo de la severidad podrían conllevar a la muerte^10^.

La problemática está en aumento dado que la TQT es considerada una medida terapéutica ineludible para el abordaje de los pacientes con VM prolongada, subestimando y normalizando las complicaciones asociadas que incrementan la estancia del paciente en la UCI, eleva los costos de la atención, disminuye la calidad de vida de los usuarios, entorpece la evolución satisfactoria en la rehabilitación y puede incluso conducirlos a la muerte^11^.

Dentro de los problemas mecánicos someramente identificados para la TQT están las complicaciones de origen mecánico o posicional, lo que se ha denominado mal-posición de la cánula TQT^12^ por causa del peso y fuerzas de tracción ejercidas por los dispositivos externos necesarios para la VM; entre éstos dispositivos están, el circuito ventilatorio, el codo tipo Swivel, el filtro nariz de camello y la succión cerrada entre otros. Adicionalmente, existen fuerzas dinámicas como la tracción generada en la movilización y los cambios de decúbito en cama de los pacientes para prevenir úlceras por presión y para realizar la higiene rutinaria.

Las complicaciones asociadas a TQT en su mayoría son prevenibles^13^, pero dependerá del conocimiento y experticia en el manejo del grupo interdisciplinar, dado que creemos que el desconocimiento sobre las complicaciones asociadas a la TQT puede aumentar la iatrogenia, el número de eventos adversos y la severidad de las complicaciones.

No es claro si la literatura científica ha abordado a las complicaciones asociadas a TQT desde un punto de vista tanto mecánico/posicional como no mecánico, debido a la poca información de calidad sobre los efectos adversos causados por el procedimiento en mención^1^, lo que limita generar soluciones para la atención en salud basadas en evidencia científica, esto establece la importancia de realizar un escaneo integrativo de la literatura científica que recopile, describa y analice la información como base para futuros estudios sobre este tema relevante para los servicios de UCI a nivel mundial.

Como antecedente de revisión se encuentra una narrativa sobre las complicaciones asociadas al procedimiento de TQT publicada en Estados Unidos por Anthony Cipriano en 2015, quien describe las diferentes complicaciones relacionadas con la TQT clasificándolas en preoperatorias y postoperatorias^14^, no obstante, hasta el momento no ha sido publicada una revisión conceptual- analítica para comparar el alcance de la información de los artículos sobre el tema en cuanto a las complicaciones de la TQT y sus factores de riesgo relacionados con aspectos mecánicos estáticos y dinámicos. Por lo anterior, para facilitar conceptualmente la presente búsqueda en consenso con los autores según la plausibilidad de la etiología de las complicaciones conocidas se realizó una clasificación de éstas en términos mecánicos posicionales (Tabla 1).

De acuerdo a todo lo anteriormente plantado nuestro estudio realizó un mapeo de la literatura científica (Scoping Review) respecto a la TQT y sus complicaciones en términos de sus factores de riesgo mecánicos y no mecánicos de pacientes adultos en UCI, incluyendo la prevalencia, la incidencia, la mortalidad y la severidad de la complicación presentada, para aportar a la profundización del conocimiento en el abordaje de pacientes ventilados mecánicamente con TQT, tanto para la praxis clínica como para futuras propuestas de investigación sobre el tema.

**Tabla 1:** Clasificación de complicaciones asociadas a la TQT según su posible origen mecánico posicional y no posicional de la cánula.

Complicaciones Asociadas a la Traqueostomía	
Posicionales	No Posicionales
Desplazamiento de la cánula	Neumotórax
Decanulación	Hemorragia/Sangrado traqueal
Traqueítis	Ruta falsa
Fístula Traqueo esofágica	Lesión de tiroides
Sangrado estomal	Lesión de nervio laríngeo
Formación de granuloma	Enfisema subcutáneo
Traqueomalacia	Incisión quirúrgica >2 centímetros
Estenosis traqueal	Paro cardiorrespiratorio
Dilatación de estoma	Infección: estomal, necrosante, neumonía

*Leyenda de tabla: elaborada por los autores*

## Materiales y Metodos

El diseño es una revisión integrativa tipo Scoping Review, la pregunta de investigación y los criterios de inclusión se establecieron para la búsqueda de los referentes bibliográficos, la información fue consultada en las bases de datos PubMed y EBSCO, se seleccionaron artículos científicos publicados entre los años 2015-2021, la estrategia PICOT (Población, Intervención, Control, Resultados y Tiempo^15)^ se empleó para definir la fórmula de búsqueda que fue construida con términos MESH^16^, “TRACHEOSTOMY, COMPLICATIONS, ADULTS, INTENSIVE CARE UNIT, PREOPERATIVE, POSTOPERATIVE”, el resultado de la fórmula final fue: (Complications) AND (tracheostomy) AND (Adult OR patient) AND (preoperative OR postoperative) AND (“Intensive care units”).

La tipología de los estudios incluidos fueron artículos originales observacionales y experimentales como cohortes, casos y controles, estudios transversales, ensayos clínicos controlados y reportes de casos, no se incluyó literatura gris como tesis de grado. Para la inclusión de los artículos y extracción de datos se tuvieron en cuenta las siguientes variables: Diseño del estudio, Autor del artículo, Año de publicación, Categoría de la revista, Tipo de complicación reportada, Mortalidad asociada a complicaciones por traqueostomía, Prevalencia/Incidencia de las complicaciones por traqueostomía reportadas, y Factores de riesgo mecánicos y no mecánicos que desencadenan las complicaciones. Se filtró por los idiomas inglés, portugués y español. Se excluyeron tipos de estudio como revisiones de tema, revisiones sistemáticas y meta-análisis para evitar medición por duplicado^17^.

Como valor agregado al mapeo de la literatura se revisó con las listas de chequeo de las guías para la evaluación de la calidad metodológica pertinentes según la tipología de cada artículo seleccionado así: CARE para Reportes de Caso, CONSORT para Ensayos Clínicos, NEWCASTLE OTAWA en su versión específica para Casos y Controles y STROBE para Cohortes, según la puntuación obtenida se clasificó y estratificó en alta calidad aquellos que obtuvieron un porcentaje de cumplimiento >80%, en calidad moderada aquellos >50% pero <79% y los de baja calidad los que eran <49%; fueron semaforizados la siguiente manera, verde alta calidad, amarillo moderada calidad y rojo los de baja calidad, se considera que los artículos con un porcentaje de cumplimiento menor al 80% tienen una probabilidad media de riesgo de sesgo y por debajo de 50% una probabilidad alta de sesgo.

Como guía metodológica y de calidad para el presente estudio se utilizó la lista de chequeo PRISMA-ScR indicada para Scoping Review de la literatura. Para evitar sesgo de notificación se presentaron todos los hallazgos encontrados en los artículos seleccionados para los resultados de la pregunta de investigación y se notificaron todas las diferencias.

En términos del objetivo y para facilitar la clasificación de los datos obtenidos respecto a las complicaciones asociadas a la TQT se propone por consenso de los autores la clasificación de éstas respecto a si su origen es mecánico (estático y/o dinámico) o no mecánico.

### Aspectos éticos

Según la resolución 8430 de 1993 del Ministerio de Salud y Protección Social en su Artículo 11, el estudio se clasifica en la categoría de investigación sin riesgo, ya que es una investigación observacional retrospectiva sin ningún tipo de intervención o modificación intencionada de las variables biológicas, fisiológicas, psicológicas o sociales. La investigación se desarrolló bajo los principios de autonomía de los artículos que se encuentran publicados, lo que otorga permiso para realizar consultas en procesos investigativos. Bajo el principio de justicia se manejaron los artículos de manera imparcial y sin ninguna predilección, este proyecto se realizó con el objetivo de aportar al desarrollo científico, fundamentar la práctica clínica y contribuir a nuevas investigaciones al respecto.

## Resultados

**Figura 1:**
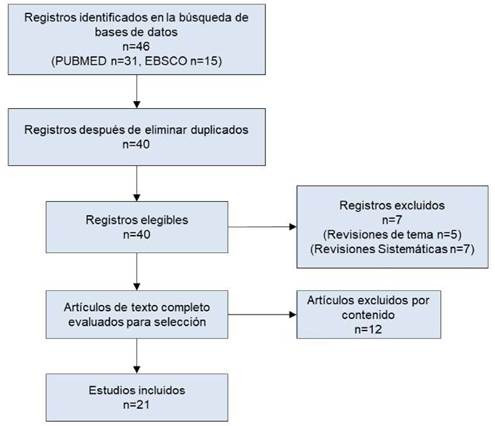
Descripción de los estudios incluidos en la Scoping Review sobre complicaciones asociadas a la TQT.

Un total de 46 artículos encontrados en las bases de datos de EBSCO y PubMed fueron elegibles de acuerdo a la ecuación de búsqueda, se eliminaron 6 artículos por duplicado, 5 por su diseño de revisión de tema y 2 por ser revisión sistemática. Después de aplicar todos los criterios de selección fueron excluidos 12 artículos (Figura 1). De los 21 artículos incluidos, el 85,7% tienen diseño tipo observacional (52,3% cohortes, 14,2% casos y controles, 9,5% series de casos clínicos, 4,7% reporte de caso clínico y 4,7% transversales), y el 14,2% diseño tipo experimental, todos de tipo ensayos clínicos controlados.

**Figura 2:**
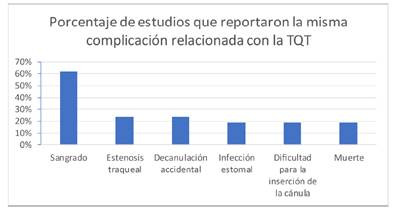
Frecuencia relativa de estudios científicos que reportaron un mismo tipo de complicación relacionada con la TQT.

Entre la gran cantidad de complicaciones encontradas en cuanto a tipo de complicación están, estenosis traqueal, infección del estoma, septicemia, sangrado, decanulación, fractura traqueal, desaturación, lesión de la pared de la tráquea, infección respiratoria, neumotórax, fistula traqueoesofágica, perforación del tubo endotraqueal, extubación espontanea, ruptura del manguito de la cánula, dificultad en la inserción de la cánula, traqueomalacia, enfisema subcutáneo, granuloma supra-estomal, obstrucción de la TQT, difícil destete, atelectasia, estenosis subglótica, fistula traqueocutánea, ruptura del anillo traqueal, daño en el balón de la cánula, lesión esofágica, neumonía asociada, desplazamiento de la cánula, parálisis de las cuerdas bucales, granuloma en la tráquea y muerte (Tabla 2), las complicaciones reportadas con mayor frecuencia por los artículos revisados se presentan en la Figura 2.

*Leyenda de figura: Adaptado por los autores de PRISMA Extension for Scoping Reviews (PRISMA-ScR18).*

**Tabla 2:** Variables de salida sobre las complicaciones mecánicas y no mecánicas relacionadas con la TQT

Autor/ Año	Revista/ Categoría	Diseño del estudio	n	Tipo de complicación / frecuencia absoluta y relativa	Mortalidad asociada a la complicación	Factores de Riesgo RMP o RNP
Yang/2018^19^	Laryngoscope Q1	Cohorte Retrospectivo	8,682	Muerte	Un índice de Charlson >2 incrementa la mortalidad P<0,05	Técnica percutánea asociada a la mortalidad OR=1,17 (RNP)
Li /2018^20^	Otolaryngology Head and Neck Surgery Q1	Cohorte Retrospectivo	1,656	Estenosis traqueal / 43 (2,59%)		Técnica Bjork flap OR=0,4 (RMP) Obesidad OR=5,15 Tubo TQT >6 OR= 2,6 (RNP)
Schaefer/ 2016^21^	ASAIO Journal Q2	Cohorte Retrospectivo	34	Infección en estoma / 2 (5,8%) Septicemia / 2 (5,8%) Muerte	A 30 días: 47,1% Al año: 11,7%	
Gigliotti / 2018^22^	Journal of Oral and Maxillofacial Surgery Q1	Cohorte Retrospectivo	111	Sangrado / 1 (0,9%) Decanulación / 1 (0,9%)	0%	
Nowak / 2017^23^	BMC Anesthesiology Q2	Cohorte Prospectivo	180	Sangrado / 10 (5,5%) Fractura traqueal / 30 (16,6%) Desaturación / 12 (6,6%) Lesión de la pared de la tráquea / 2 (1,1%)		
Affronti / 2019^24^	Journal of Cardiothoracic and Vascular Anesthesia Q2	Cohorte Retrospectivo	5,148	Infección respiratoria / 2 (0,03%) Sangrado / 11 (0,21%) Neumotórax / 1 (0,01%) Fistula Traqueoesofágica / 1 (0,01%) Estenosis traqueal / 1 (0,01%) Decanulación / 1 (0,01%)	35,7% durante la hospitalización 40,2% después de 3 meses del alta 64,3% al año o dos años del alta	
Sahiner/ 2017^25^	Medical Science Monitor Q2	Ensayo Clínico	78	Sangrado / 22 (28,2%) Perforación del tubo endotraqueal / 8 (6,6%) Decanulación / 3 (3,3%)	1,2% (1 de los 78 pacientes intervenidos)	
Saritas / 2016^26^	JPMA Q3	Ensayo Clínico	60	Extubación espontanea / 6 (10%) Sangrado / 5 (8,3%) Ruptura del manguito de la cánula / 5 (8,3%)		
Xaplanteri / 2019^27^	Respiratory Case Reports	Reporte de caso	1	Estenosis traqueal / 1 (100%)		
Karimpour / 2017^28^	Archives of Iranian Medicine (AIM) Q2	Transversal	184	Estenosis traqueal / 3 (1,8%) Infección del estoma / 4 (2,5%) Dificultad en inserción de cánula / 4 (2,5%) Fistula traqueoesofágica / 2 (1,2%) Traqueomalacia / 1 (0,6%)		
Alabi /2018^29^	Annals of African Medicine Q3	Casos y Controles	76	Enfisema subcutáneo / 4 (5,2%) Granulación supraestomal / 3 (3,9%) Sangrado / 2 (2,6%) Decanulación / 2 (2,6%) Estenosis traqueal / 2 (2,6%) Obstrucción de la TQT / 1 (1,3%) Difícil destete / 1 (1,3%)	En 15% de los casos incluidos	
Autor/ Año	Revista/ Categoría	Diseño del estudio	n	Tipo de complicación / frecuencia absoluta y relativa	Mortalidad asociada a la complicación	Factores de Riesgo RMP o RNP
Araujo/ 2015^30^	Medicina intensiva Q2	Cohorte Prospectivo	70	Sangrado / 10 (14,2%) Atelectasia / 3 (4,2%) Dificultad en inserción cánula / 9 (14,2%) Perforación del tubo endotraqueal / 2 (2,8%)		
Ahmed/ 2018^31^	Pakistan Armed Forces Medical Journal NR*	Ensayo Clínico	534	Sangrado / 8 (1,49%) Decanulación / 2 (0,37%) Estenosis subglótica / 4 (0,74%) Fistula Traqueoesofágica / 4 (0,74%) Fistula traqueo- cutánea / 2 (0,37%)	0,37% de los pacientes incluidos	
Araujo/ 2018^8^	Medicina intensiva Q2	Cohorte Prospectivo	114	Sangrado / 23 (20%) Desaturación / 2 (1,7%) Atelectasia / 4 (3,5%) Dificultad en la inserción de la cánula / 19 (16,6%) Ruptura de anillo traqueal / 1 (0,8%) Daño del balón de la cánula / 2 (1,7%) Estenosis traqueal / 2 (1,7%)	44,7% de los pacientes incluidos 19,6% en TQT de urgencia 17,6% Por paro cardiorrespiratorio 15,6% por neumonía asociada.	
Gan / 2020^32^	Journal of Medical Sciences Q1	Casos y Controles	140	Estenosis traqueal / 14 (10%) Enfisema subcutáneo / 17 (12,1%) Septicemia / 17 (12,1%) Sangrado / 8 (5,7%)		
Royo / 2015^33^	Surgical Spanish Q3	Casos y Controles	291	Sangrado / 30 (2,7%) Infección en estoma / 17 (1.6%) Neumotórax / 5 (0,46%) Atelectasia / 33 (31%) Lesión esofágica / 4 (0,37%) Neumonía asociada / 26 (2,4%)	82% de los pacientes incluidos	
Meier / 2019^34^	BMC anesthesiology Q2	Series de Casos Clínicos	47	Neumonía asociada / 1 (2,1%)		
Young / 2017^35^	Oral Surgery, Oral Medicine, Oral Pathology and Oral Radiology Q2	Series de Casos Clínicos	115	Sangrado / 4 (4,5%) Difícil inserción de cánula / 1 (0,86%)		
Hee / 2018^36^	World journal of Surgery Q1	Cohorte Longitudinal	236	Sangrado / 5 (2,1%) Infección en estoma / 17 (7,2%) Desplazamiento de la cánula / 5 (2,1%) Parálisis de las cuerdas bucales / 4 (1,6%) Granuloma en tráquea / 4 (1,6%)	9,7% de los pacientes incluidos	
Tebano / 2016^37^	Transplant Infectious Disease Q2	Cohorte Retrospectivo	176	Infección Muerte	24% en UCI 13% al día 28 post- TQT 29% al año	Infección OR=3,28 (RNP)
Parsikia / 2016^38^	Journal of Surgical Research NR*	Cohorte Retrospectivo	500	Muerte	9.8% post-TQT	Ventilación mecánica preoperatoria OR=3,14 (RNP)

Leyenda de tabla: *NR: No Reporta. RMP: Riesgo Mecánico Posicional, RNP: Riesgo No Posicional.

*Leyenda de tabla: las complicaciones con mayor frecuencia de reporte fueron sangrado 61,9% (13/21 artículos), estenosis traqueal 23,8% (5/21 artículos), decanulación 23,8% (5/21 artículos), infección de la estoma 19% (4/21 artículos), dificultad en la inserción de la cánula 19% (4/21 artículos) y muerte 19% (4/21).*

La incidencia por complicación osciló para sangrado entre 0,9% y 28,2%, mientras que la estenosis traqueal osciló entre 0.01%-10%, la decanulación entre 0,01%-3,3%, la infección de la estoma entre 1,6% -7,2%, y la dificultad en la inserción de la cánula varió entre 0,86% -16,6%.

Yang y cols, en el estudio que realizaron en 2018 demuestran que un índice de Charlson >2 incrementa la mortalidad asociada a la TQT con una p<0,05, por otro lado, Schaefer y cols., en el 2016 no registran casos de muerte durante el procedimiento de la traqueostomía, pero sí reportan altas tasas de mortalidad a los 30 días y al año posterior a la intervención. Affronti y cols, en el 2018 reportaron muertes en un 40,2% después de 3 meses de alta y 64,3% al año o dos años de la inclusión de los pacientes en el estudio, otro estudio que reporta altas tasas de mortalidad fue el realizado por Tebano y cols., en 2016, reportando un 24% de mortalidad en UCI, 13% a los 28 días en el servicio de hospitalización y 29% al año en los pacientes ambulatorios.

En relación a los factores de riesgo no posicionales/mecánicos asociados a la TQT se identificaron la obesidad con un OR de 5,15, estar en VM previa a la TQT con un OR=3,14, un diámetro de tubo de TQT >6 con un OR= 2,6 y finalmente, la técnica percutánea se asoció de manera limítrofe a mayor mortalidad con un OR=1,17. En términos de valor de p se encontró que un número de plaquetas disminuido predispone a complicación peri-operatoria (p<0,001).

Pocos estudios reportaron factores de riesgo mecánicos o posicionales para desarrollar complicaciones relacionadas con la TQT de especial interés en este mapeo de la literatura, sólo Li y cols., plantearon que la Técnica Bjork flap puede ser un factor protector con un OR=0,4, debido a que proporciona estabilidad mecánica a la TQT durante la tos, el estornudo y los cambios de posición.

Como valor agregado se realizó una evaluación a la calidad de los artículos seleccionados encontrando que el 47,6% se clasifican en alta calidad debido a que cumplen con el >80% de los ítems de lasescalas de calidad pertinentes a la tipología de diseño de cada estudio, como de moderada calidad el 38% y como de baja calidad el 14,2%, entendiéndose que a menor calidad mayor riesgo de sesgo (Ver Tabla 3).

**Tabla 3:** Clasificación de la calidad de los estudios incluidos.

Autor	Año	Escala	Cumplimiento de criterios	Calificación
Gan^32^.	2020	NEWCASTLE	8/8	100
Li^20^.	2018	STROBE	21/22	95
Gigliotti^22^.	2018	STROBE	21/22	95
Meier^34^.	2017	CARE	12/13	92
Hee^36^.	2018	STROBE	20/22	91
Affronti^24^.	2018	STROBE	20/22	91
Parsikia^38^.	2016	STROBE	19,5/22	89
Yang^19^.	2018	STROBE	19/22	86
Young^35^.	2017	CARE	11/13	85
Karimpour^28^.	2017	CONSORT	18/25	82
Araujo^8^.	2018	STROBE	17/22	77
Xaplanteri^27^.	2019	CARE	10/13	76
Araujo^30^.	2015	STROBE	16/22	73
Schaefer^21^.	2016	STROBE	14,5/22	66
Nowak^23^.	2017	STROBE	14/22	64
Tebano^37^.	2016	STROBE	14/22	64
Alabi^29^.	2018	NEWCASTLE	5/8	63
Saritas^26^.	2016	CONSORT	14,5/25	58
§ahiner^25^.	2017	CONSORT	11/25	44
Ahmed^31^.	2018	CONSORT	8/25	32
Royo^33^.	2015	NEWCASTLE	2/8	25

*Leyenda de tabla: se realizó una semaforización de acuerdo al porcentaje de cumplimiento en las listas de chequeo de las guías para evaluación de la calidad pertinentes al diseño del estudio reportados así: verde alta calidad con >80% de cumplimiento de los ítems evaluados, amarillo moderada calidad con <79% y >50% de cumplimiento, rojo de baja calidad con <49% de cumplimiento.*

## Discusión

En este estudio se realizó un mapeo mediante un Scoping Review de la literatura científica disponible en bases de datos de EBSCO y Pubmed según los criterios de selección establecidos, donde se reportaron las complicaciones asociadas a la TQT en pacientes adultos en las unidades de cuidados intensivos entre el 2015-2020. Entre las complicaciones con mayor incidencia en los artículos de mejor calidad fueron, muerte, sangrado, infección en estoma con un 33% cada una, igualmente para estenosis traqueal, decanulación, fistula traqueoesofágica un 22% cada una, y en un 11% complicaciones como septicemia, infección respiratoria, neumotórax, dificultad en la inserción de la cánula, traqueomalacia, neumonía, desplazamiento de la cánula y parálisis de las cuerdas bucales.

En la presente revisión se encontró que la obesidad es un factor predisponente para el desarrollo de complicaciones como la estenosis traqueal con un OR de 5,15^20^, lo que se relaciona con lo reportado en el estudio de Halum y cols., donde reportan a la obesidad como factor de riesgo significativo para el desarrollo de estenosis con una prevalencia de 9.9% en pacientes obesos con IMC >30, comparado con el 0.4% en pacientes sin obesidad^39^.

En comparación con la literatura científica disponible no se encontraron artículos con la misma pregunta de investigación, el estudio con mayor similitud fue en una revisión sistemática publicada por Meng y cols., en 2015 con el objetivo de evaluar variables como mortalidad, duración de la VM, duración de la sedación, duración de la estancia en la UCI e incidencia de neumonía asociada a la TQT. En los resultados del estudio encontraron que 26,9% de los pacientes (273/1018) murieron en el grupo de TQT temprana y 30,5% (312/1021) pacientes murieron en el grupo TQT tardía sin encontrar diferencias estadísticamente significativas^40^. Al compararlo con los datos de mortalidad encontrados en la presente revisión, se evidencia mayor variabilidad oscilando entre 0,37% y 64%, con una media de 22,7% cercana a la reportada por el autor mencionado.

En la literatura publicada respecto a los factores mecánicos estáticos y dinámicos relacionados con la TQT sólo encontramos un autor, Schimth (2008), quien identifica a la mal-posición de la cánula como factor de riesgo mecánico, reportando que de 403 pacientes evaluados el 10% presentaba mala alineación de la cánula TQT y que esto se asociaba a la VM prolongada^12^. Es importante resaltar que a nivel nacional Quintero y cols.^41^ en un estudio no publicado encontraron mal-posición de la TQT en un 80% de los pacientes en la UCI, lo que sugiere que es urgente disminuir el sub-reporte de esta condición en los servicios de UCI y hospitalización para plantear estrategias correctivas conducentes a prevenir complicaciones resultantes de aspectos posicionales estáticos y dinámicos como son, obstrucción de la cánula (muy frecuente en nuestra praxis clínica), formación de tejido de granulación, fístula traqueoesofágica, estenosis traqueal, desplazamiento externo de la cánula, decanulación accidental, traqueítis, sangrado y traqueomalacia^12^^,^^41^.

Por lo anterior, la pregunta relacionada con los factores de riesgo mecánicos o posicionales no pudo ser resuelta en la presente revisión de alcance de la literatura, siendo una limitación no dar respuesta a la variable de interés debido a un claro sub-registro de un problema clínico común en el día a día como es la variabilidad posicional de las cánulas de TQT expuestas a la tracción por el peso del circuito ventilatorio entre otros dispositivos y por los cambios de decúbito en cama necesarios para posicionamiento o para las actividades de aseo^41^. Sólo un dato de factor protector mecánico fue identificado por Li y cols., quienes defienden que la técnica Bjork Flap proporciona estabilidad a la TQT durante la tos, el estornudo y los cambios de posición con un OR=0,4^20^.

Otra de las limitaciones de nuestro estudio fue relacionada con el número de bases de datos consultadas con acceso libre remoto EBSCO y PubMed en tiempos de pandemia, no obstante, son bases de datos robustas que pueden condensar entre ellas la mayoría de artículos científicos disponibles en los idiomas seleccionados.

Como antecedentes, diversos autores que han publicado datos previos a nuestra ventana de revisión de 2015-2020, han reportado complicaciones peri y postoperatorias relacionadas con la TQT, entre estas están, sangrado (75%42), neumonía nosocomial (26%),^44^, estenosis traqueal (12%),^46^, infección en estoma (5%5), enfisema subcutáneo 1.4%^47^, fistula traqueoesofágica (>1%), muerte perioperatoria (0.16%) asociadas con TQT percutáneas^39^, neumotórax (17%48) y desplazamiento de la cánula (de 0.35 a 2.6%). En comparación con los resultados de nuestro estudio las incidencias mencionadas difieren de la siguiente manera, sangrado 61%, estenosis traqueal 28,5%, infección en estoma 19%, mortalidad 19%, dificultad en la inserción de la cánula 19% y decanulación accidental 23,6%.

La principal fortaleza del presente estudio consiste en aportar identificación del alcance de la evidencia científica actualizada mediante un Scoping Review en el periodo evaluado respecto a las complicaciones asociadas a la TQT desde un concepto posicional o mecánico, esto permite conocer los datos disponibles que podrían ser evaluados a mayor profundidad y con análisis estadísticos pertinentes en una revisión sistemática con meta-análisis.

Otro concepto importante que proponemos como problema cotidiano, son las constantes fuerzas mecánicas tanto estáticas como dinámicas que soporta la TQT y que generan mala alineación, fricción, puntos de presión sobre el tejido traqueal y lesiones tisulares relacionadas, pero que al momento no han sido debidamente evaluadas ni publicadas en artículos científicos, persistiendo sin solución en un contexto de vida real erróneamente normalizado y aceptado, esto impide plantear propuestas innovadoras y estrategias preventivas para las complicaciones de origen posicional relacionadas con la TQT en los diferentes niveles de atención en salud de todas las UCI.

Otro aspecto a discutir es que debido a que la pregunta de investigación no se centraba en los tipos de técnicas quirúrgicas de TQT, estas no fueron incluidas en la ecuación de búsqueda lo que genera una limitación que sólo fue identificada durante la lectura del texto de los artículos como el de Yang y cols., donde reportan que la técnica percutánea se asocia a mayor mortalidad con un OR limítrofe de 1,2^19^, permaneciendo controversial cuál técnica entre la quirúrgica y la percutánea es conveniente para el paciente de UCI. La literatura ha planteado distintas posiciones como la de Bacchetta y cols.^50^, quienes reportan que el 42% de complicaciones encontradas en su estudio estaban relacionadas con la técnica quirúrgica y sólo un 12% con la percutánea. Por otro lado, Celedón y cols.^51^, encontraron que dependiendo del tiempo quirúrgico se presentaban diferencias en la incidencia de complicaciones, para el tiempo peri-operatorio reporta complicaciones relacionadas con la técnica percutánea con un 40% y en la quirúrgica 16%, contrario al tiempo post-operatorio en el que la incidencia de complicaciones con la técnica percutánea fue del 12% y la técnica quirúrgica 8%.

En la actualidad se observa una tendencia a preferir la técnica percutánea debido a que reduce los costos, disminuye el uso de quirófanos y equipos necesarios para realizar el procedimiento50, sin embargo, lo anterior depende en gran medida del entrenamiento y experticia de los profesionales a _cargo_^52^^,^^53^_._

Entre las preguntas que siguen sin ser resueltas para el desarrollo de estudios futuros, están interrogantes como ¿sería pertinente realizar estudios sobre la prevalencia de la mal-posición y demás factores mecánicos/posicionales de la cánula de TQT en los servicios de UCI? ¿Existen estrategias o dispositivos para disminuir la incidencia de complicaciones de origen mecánico relacionadas con la TQT?

Se recomienda con esta investigación que se lleven a cabo estudios observacionales en donde se identifique si las fuerzas mecánicas y estáticas que soporta la TQT generan algún tipo de complicación y posteriormente estudios experimentales en los que se evalúen estrategias o dispositivos para prevenir y corregir el mal-alineamiento o mal-posicionamiento de la cánula de TQT como desencadenante de complicaciones intra y extra-traqueales que en conjunto enriquezcan la evidencia científica disponible y el abordaje del paciente con TQT en VM.

## Conclusiones

En el presente Scoping Review de la literatura científica se logró identificar que la TQT aunque es una técnica terapéutica muchas veces ineludible también tiene complicaciones asociadas a la misma como son el sangrado, la estenosis traqueal y la decanulación accidental entre otras que pueden conducir incluso a la muerte, sin embargo, aún se desconoce en definición conceptual y en evidencia si proceden de una causa mecánica o no mecánica, lo cual consideramos un conocimiento relevante para direccionar decisiones y estrategias en el abordaje de pacientes con TQT durante la VM en las UCI, con el objetivo de lograr disminuir la incidencia de complicaciones asociadas y reportadas hasta el momento.
